# Neuroprotective Terpenoids Derived from *Hericium erinaceus* Fruiting Bodies: Isolation, Structural Elucidation, and Mechanistic Insights

**DOI:** 10.3390/ijms26146606

**Published:** 2025-07-10

**Authors:** Ying Cao, Qiaona Wang, Lu Li, Haitao Jiang, Bianjiang Zhang, Yulong Wu, Feng Zhou, Chun Hua, Guangming Huo, Shengjie Li, Jianmei Li

**Affiliations:** 1 School of Food Science and Pharmaceutical Engineering, Nanjing Normal University, Nanjing 210023, China; 232702036@njnu.edu.cn (Y.C.); 232702032@njnu.edu.cn (L.L.); 2 School of Food Science, Nanjing Xiaozhuang University, Nanjing 211171, China; 2005026@njxzc.edu.cn (H.J.); zhangbjiang@njxzc.edu.cn (B.Z.); wuyl_8080@163.com (Y.W.); zhoufeng@njxzc.edu.cn (F.Z.); hc3501988@163.com (C.H.); cnhuogm@njxzc.edu.cn (G.H.); 3 School of Ecology and Applied Meteorology, Nanjing University of Information Science & Technology, Nanjing 210044, China; 202311530005@nuist.edu.cn

**Keywords:** *Hericium erinaceus*, transcriptomics, molecular docking, bioactive compounds, antioxidants, oxidative stress

## Abstract

*Hericium erinaceus*, a medicinal macrofungus, is renowned for its potential neuroprotective benefits. Here, we isolated and characterized secondary metabolites from *H. erinaceus* fruiting bodies and explored their neuroprotective effects and primary mechanisms of action. A novel terpenoid (4) and four known compounds (**1**, **2**, **3**, and **5**) were identified. Their chemical structures were determined using nuclear magnetic resonance (NMR), high-resolution mass spectrometry (HRMS), and x-ray diffraction (XRD). Bioactivity screening using PC12 cells indicated that (3R,4R)-4-acetyl-3,4-dihydro-6,8-dihydroxy-3-methoxy-5-methyl-1H-2-benzopyran (3) and the terpenoid, (1R,4S,8aS)-1,4-dihydroxy-2,5,5,8a-tetramethyl-1,4,4a,5,6,7,8,8a-octahydronaphthalene-1-carbaldehyde (4), demonstrated protective properties against hydrogen peroxide (H_2_O_2_)-induced damage. Transcriptomics, network pharmacology, and molecular docking showed that compound **4** counteracted H_2_O_2_-induced oxidative stress and inflammation by substantially attenuating pro-inflammatory cytokine (IL-1β, IL-6) expression, downregulating pro-oxidant factors (Aoc3, Dusp3), and decreasing reactive oxygen species levels, while boosting superoxide dismutase activity. Compound **4** exerted neuroprotective effects via the NF-κB pathway. *H. erinaceus* represents a valuable natural reservoir of bioactive compounds for treating and preventing neurodegenerative diseases.

## 1. Introduction

*Hericium erinaceus* (Bull.: Fr) Pers. (Agaricales, Russulales, Hericaceae) is an edible, medicinal macrofungus predominantly found in the mountainous regions of Asia. It is widely used in traditional medicine and cuisine because of its high nutritional and medicinal value [1,2]. *H. erinaceus* is a mesophilic and hygrophilic fungus thriving under low-light conditions on decaying or fallen broadleaf tree trunks. The fungus has been used for more than two millennia to treat digestive system disorders [3,4,5]. Modern research indicates that the active components of *H. erinaceus* demonstrate hepatoprotective, gastroprotective, hypoglycemic, neuroprotective, anti-aging, and antioxidant properties [6,7,8,9,10].

The fruiting bodies and mycelia of *H. erinaceus* are abundant in various chemical components. Various bioactive compounds have been identified to date in *H. erinaceus*, including polysaccharides, phenolics, steroids, alkaloids, and terpenes [11]. For instance, Qiao et al. [12] characterized an alkali-soluble β-glucan (AHEP-A-b) purified from alkali-extracted polysaccharides of *H. erinaceus* fruiting bodies whose main backbone is composed of (1→6)-linked-D-β-glucopyran residues. AHEP-A-b displays differential scavenging efficiencies against different reactive oxygen species (ROS), demonstrating its antioxidant activity. Lin et al. [13] obtained a phenolic compound with an isoindolinone skeleton from *H. erinaceus* mycelia termed Erinacerin W that exhibited immunomodulatory and neuroprotective effects. Moreover, Li et al. [14] characterized four novel sterols from *H. erinaceus* fruiting bodies, of which the erinarols H and J markedly suppressed tumor necrosis factor (TNF)-α and nitric oxide (NO) production in lipopolysaccharide-stimulated mouse macrophages, suggesting their potential for treating inflammatory diseases. Song et al. [15] reported the isolation of a novel alkaloid and 4-hydroxy aldehyde derivative from *H. erinaceus* mycelia, which exhibited potential antifungal biofilm-forming activity. Recently, Wei et al. [16] isolated and characterized two new diterpenoids from *H. erinaceus* as follows: a verrucosane diterpene and a cyathane-xyloside containing an unconventional hemiacetal moiety. Despite advances in the isolation and identification of natural products from *H. erinaceus*, systematic research on their mechanisms of action is limited, impending their further development and industrial application [17].

*Hericium erinaceus* has garnered growing interest owing to its therapeutic potential in addressing neurological disorders. This neuroprotective activity is primarily attributed to the bioactive small molecules within *H. erinaceus*, which operate through anti-oxidative, anti-inflammatory, and neurotrophic mechanisms, stimulating and promoting nerve growth factor (NGF) synthesis. Tzeng et al. [18] purified diterpenoid, erinacine A, meroterpenoid, and erinacine S, from *H. erinaceus* fruiting bodies. They revealed that erinacine A and S reduced amyloid plaque formation and enhanced hippocampal neurogenesis in a murine model of APP/PS1 Alzheimer’s disease (AD) by suppressing neuroinflammation and upregulating the expression of insulin-degrading enzymes, highlighting their therapeutic potential in AD. Zhang et al. [19] discovered a novel dihydropyridine derivative and two known compounds in *H. erinaceus* mycelia via methanol extraction. These compounds enhanced NGF-induced neurite outgrowth and safeguarded differentiated PC12 cells from NGF deprivation via TrkA-mediated and Erk1/2-dependent pathways. In parallel, Zhang et al. [20] isolated three novel terpenoids, erinacines T–V, from *H. erinaceus* liquid cultures that displayed pronounced neuritogenic effects in PC12 cells, thus proposing novel candidates for neurotrophic drug development against neurodegenerative diseases.

PC12 cells, as a classical neuronal model, are widely utilized in research on neuronal injury, neuroprotection, and neurodegenerative diseases and play a crucial role in neuroscience research [21]. However, because of the chemical complexity of *H. erinaceus* and the current limitations in functional investigations on its bioactive components, research should prioritize the isolation, identification, and mechanistic elucidation of its active constituents to clarify the basis of its efficacy [22].

To explore the health benefits of *H. erinaceus* and identify compounds with strong bioactivity and diverse structures, this study delved deeper into the efficacy and mechanisms of the action of newly isolated compounds on neurological disorders using a PC12 cell model. We further aimed to advance foundational research on *H. erinaceus* by investigating the functional activities of a terpenoid compound isolated from its fruiting bodies and providing initial mechanistic insights into its neuroprotective effects. These findings establish a critical research foundation for the extraction, processing, and valorization of antioxidant bioactive constituents from *H. erinaceus* underpinning the potential application of this macrofungus and its extracts as novel nutraceutical ingredients in antioxidant-rich functional foods.

## 2. Results

### 2.1. Structural Elucidation of Compounds ***1***–***5***

**Figure 1:**
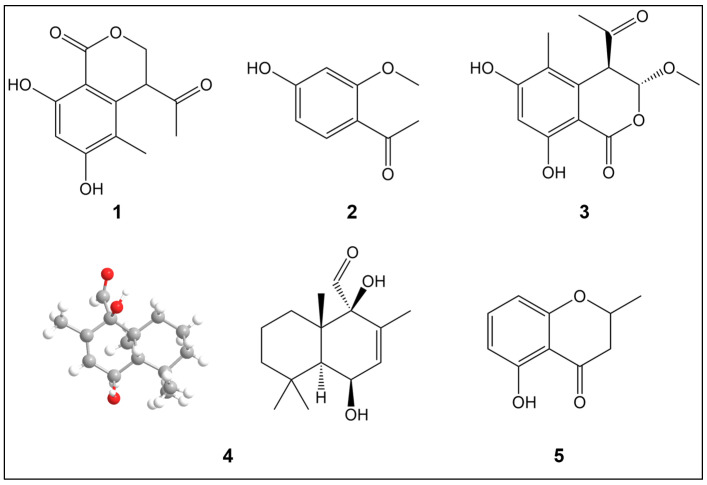
Structures of compounds **1**–**5** derived from *Hericium erinaceus*. Color code: oxygen (red), carbon (gray), hydrogen (white).

A novel terpenoid, (1R,4S,8aS)-1,4-dihydroxy-2,5,5,8a-tetramethyl-1,4,4a,5,6,7,8,8a-octahydronaphthalene-1-carbaldehyde (4), along with four known phenolic compounds, was isolated from the ethanol extract of *H. erinaceus* fruiting bodies. The known compounds were identified as 4-acetyl-3,4-dihydro-6,8-dihydroxy-5-methyl-1H-2-benzopyran-1-one (1), 1-(4-hydroxy-2-methoxyphenyl)ethenone (2), (3R,4R)-4-acetyl-3,4-dihydro-6,8-dihydroxy-3-methoxy-5-methyl-1H-2-benzopyran (3), and 2,3-dihydro-5-hydroxy-2-methyl-4H-1-benzopyran-4-one (5) (Figure 1).

Compound **4** was isolated as a white powder. The molecular formula was determined as C_15_H_24_O_3_ based on the HREIMS peak at *m*/*z* 275.16185 [M + Na]^+^, indicating three degrees of unsaturation. The infrared (IR) spectrum (KBr pellet) showed characteristic absorption bands at νmax 3338, 2944, 2832, 2522, 1450, 1030, and 672 cm^−1^. The ^1^H-NMR spectrum indicated four methyls, corresponding to three aliphatic methyl groups [δ 1.12 (3H, s), 1.18 (3H, s), and 1.29 (3H, s)] and one olefinic methyl [δ 1.60 (3H, d, *J* = 1.7 Hz)], as well as one olefinic proton [δ 4.28 (1H, dt, *J* = 10.2, 2.4 Hz)] and three methylenes [δ 1.46 (1H, m), 1.50 (1H, m), 1.54 (1H, m), 1.72 (1H, m), 1.28 (1H, m), 1.37 (1H, m), 1.64 (1H, d, *J* = 2.4 Hz), 5.83 (1H, dq, *J* = 10.2, 1.7 Hz)], along with one -CHO group. The ^13^C-NMR spectrum displayed 15 carbon resonances, corresponding to four methyl groups, three methylenes, three methines, and four quaternary carbons. Apart from one degree of unsaturation occupied by a double bond, the remaining two degrees of unsaturation indicated that compound **4** contains a bicyclic core–ring system (Table 1). Collectively, these data indicate that compound **4** is a drimane sesquiterpene. The NMR spectra suggest that compound **4** has a similar molecular scaffold to that reported for albrassitriol [23], except that a CHO replaced one of the CH_2_OH-11 groups, a structural assignment further confirmed by ^1^H–^1^H correlation spectroscopy (COSY) and heteronuclear multiple bond connectivity (HMBC) correlations (The related NMR spectra can be seen in Figures S1–S6). The absolute configuration was assigned as 6S,10R based on low-temperature (100 K) Cu Kα X-ray diffraction data (Figure 1).

### 2.2. Protective Effect of Derived Compounds on H_2_O_2_-Induced Damage

**Figure 2:**
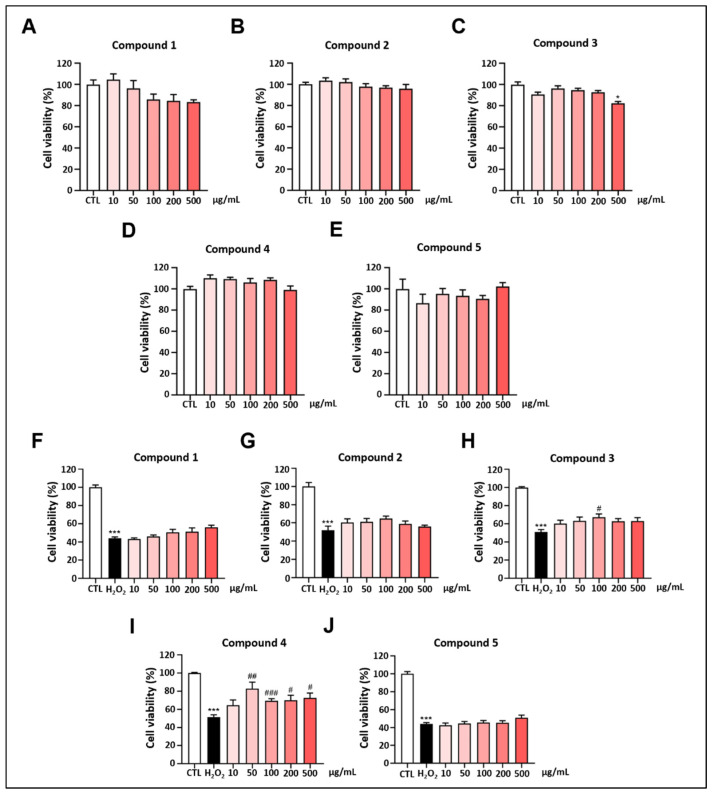
(**A**–**E**): Effects of the compounds on PC12 cell viability. (**F**–**J**): Effects of the compounds on PC12 cell viability under H_2_O_2_-induced (1000 μM) oxidative stress injury. Values are represented as mean ± standard error of mean. * Indicates significant difference (* *p* < 0.05, *** *p* < 0.001) between control and H_2_O_2_ or compound–H_2_O_2_ groups. # represents a significant difference (# *p* < 0.05, ## *p* < 0.01, ### *p* < 0.001) between H_2_O_2_ and compound–H_2_O_2_ groups.

The effects of the five compounds on PC12 cell viability were evaluated using a CCK-8 assay. The viability of PC12 cells exhibited no substantial changes compared to the control group following treatment with compounds **1**–**5** at concentrations of 10, 50, 100, 200, and 500 μg/mL (Figure 2A–E). This indicates that, within the selected concentration range, none of the compounds exhibited toxicity toward PC12 cells, except for compound **3**, which exhibited strong toxicity at 500 μg/mL.

The protective effects of the five compounds against H_2_O_2_-induced cytotoxicity in PC12 cells were assessed using a CCK-8 assay. The 1000 μM H_2_O_2_ model group exhibited reduced viability versus controls (*p* < 0.001) (Figure 2F–J). However, pretreatment with compounds **1**–**5** at concentrations of 10, 50, 100, 200, and 500 μg/mL (at 100 µg/mL for compound **3** and 50–500 µg/mL for compound **4**) enhanced cell survival rates (*p* < 0.05) compared to the model group. These findings suggest that compound **3** at 100 μg/mL and compound **4** at 50–500 μg/mL exert potent protective effects against H_2_O_2_-induced cell damage. Compound 3 was identified as 4-acetyl-3,4-dihydro-6,8-dihydroxy-3-methoxy-5-methyl-1H-2-benzopyrrole by comparing its ^1^H NMR spectra with the database, and its effects have previously been investigated [24].

In contrast, the structure and function of compound **4** have not been previously reported; therefore, we further investigated the mechanism underlying its protective effect on H_2_O_2_-induced injury, as this has not yet been determined.

### 2.3. Protective Effect of Compound ***4*** on H_2_O_2_-Induced Nerve Injury

**Figure 3:**
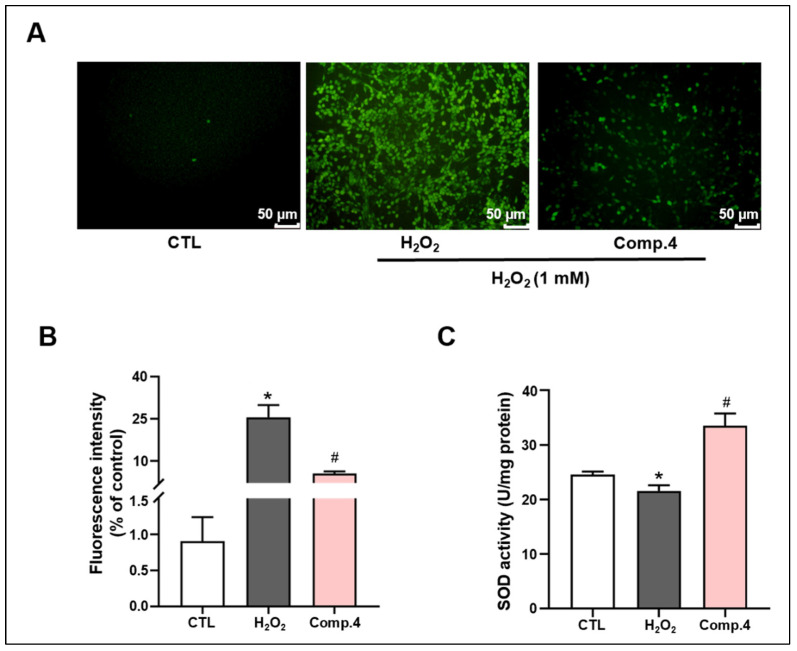
The antioxidative effect of compound **4** treatment on PC12 cells. (**A**) Representative images of reactive oxygen species (ROS) captured (scale bar: 50 μm). (**B**) Quantitative analysis of intracellular ROS levels by using Image J software. (**C**) Superoxide dismutase (SOD) in treated groups. CTL, control group; H_2_O_2_, H_2_O_2_-treated group; compounds, compound-treated group. * Indicates significant difference (* *p* < 0.05) between control and H_2_O_2_ groups. # represents a significant difference (# *p* < 0.05) between H_2_O_2_ and compound **4**–H_2_O_2_ groups.

The analysis of ROS fluorescence intensity in PC12 cells indicated that intracellular ROS levels were substantially increased in the H_2_O_2_ model compared to the control group (Figure 3A,B). In contrast, treatment with compound **4** reduced intracellular ROS levels compared to those in the model group (*p* < 0.05). Concurrently, superoxide dismutase (SOD) activity was markedly reduced by H_2_O_2_ exposure versus controls (Figure 3C). However, treatment with compound **4** substantially enhanced intracellular SOD activity compared to that in the model group. These results demonstrate compound **4**’s efficacy in countering H_2_O_2_-induced oxidative stress through inhibiting intracellular ROS production and enhancing SOD activity.

### 2.4. Transcriptomics Analysis of the Mechanisms of H_2_O_2_-Induced Neurological Injury in PC12 Cells

**Figure 4:**
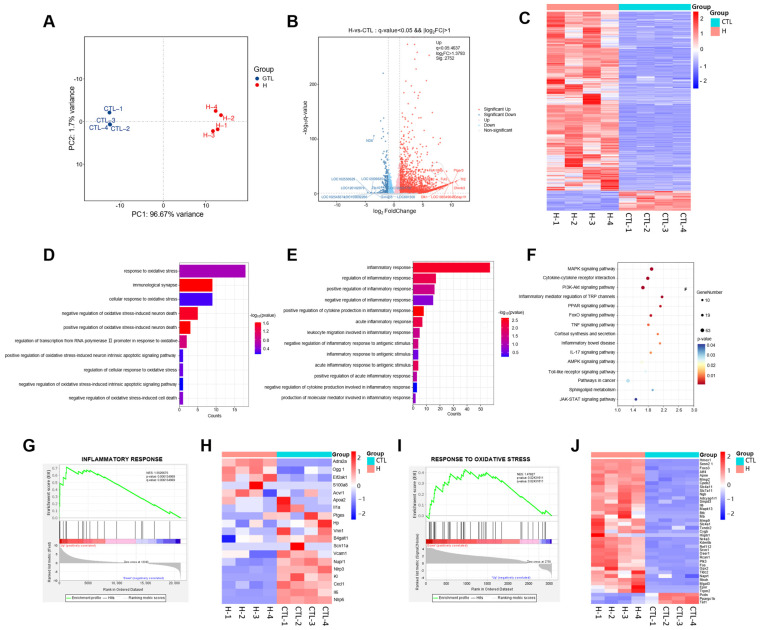
Transcriptomic analysis of differentially expressed genes (DEGs) in H_2_O_2_-treated cells. (**A**) Principal component analysis (PCA) plot of DEGs. (**B**) Volcano plot of DEGs. (**C**) Heatmap for clustering analysis of DEGs. (**D**,**E**) Gene Ontology (GO) enrichment analysis related to oxidative stress and inflammation. (**F**) Kyoto Encyclopedia of Genes and Genomes (KEGG) pathway enrichment analysis related to oxidative stress and inflammation. (**G**–**J**) Gene Set Enrichment Analysis (GSEA) enrichment analysis related to oxidative stress and inflammation.

To systematically explore the effects of H_2_O_2_ induction on gene expression in PC12 cells, RNA-seq-based transcriptomic analysis was performed on the control and H_2_O_2_-treated groups. Principal component analysis (PCA) revealed a clear segregation between the control and H_2_O_2_ groups, further supported by high intragroup consistency (Figure 4A). This analysis identified a total of 3061 differentially expressed genes (DEGs), comprising 2752 upregulated and 309 downregulated genes. The DEGs were graphically represented by volcano plots and heat maps (Figure 4B,C).

Gene Ontology (GO) enrichment analysis yielded 584 terms. Figure 4D,E highlight the top 10 significantly enriched terms associated with oxidative stress and the top 13 terms related to inflammation, including cellular response to oxidative stress, positive regulation of oxidative stress-induced neuronal death, production of molecular mediators involved in inflammatory responses, and regulation of inflammatory responses. To further elucidate the signaling pathways associated with the DEGs, a KEGG pathway analysis was performed. In total, 64 signaling pathways were enriched, with pathways linked to oxidative stress and inflammation being notably altered. For clarity, fifteen of these pathways were selected for visualization, including IL-17, TNF, and Toll-like receptor (TLR) signaling pathways (Figure 4F).

Gene set enrichment analysis (GSEA) was performed to identify signaling pathways altered by H_2_O_2_ in PC12 cells. GSEA indicated marked alterations in the gene sets associated with oxidative stress and inflammatory responses following H_2_O_2_ stimulation (Figure 4G–J). Pro-inflammatory factors and pro-oxidant genes were upregulated, whereas anti-inflammatory cytokines and antioxidant factors were downregulated. Collectively, these findings suggest that the neuronal damage model induced by H_2_O_2_ was primarily mediated by oxidative stress and inflammation.

### 2.5. Interaction Analysis of Compound ***4*** and Oxidative Stress and Inflammation-Related Target Genes

**Figure 5:**
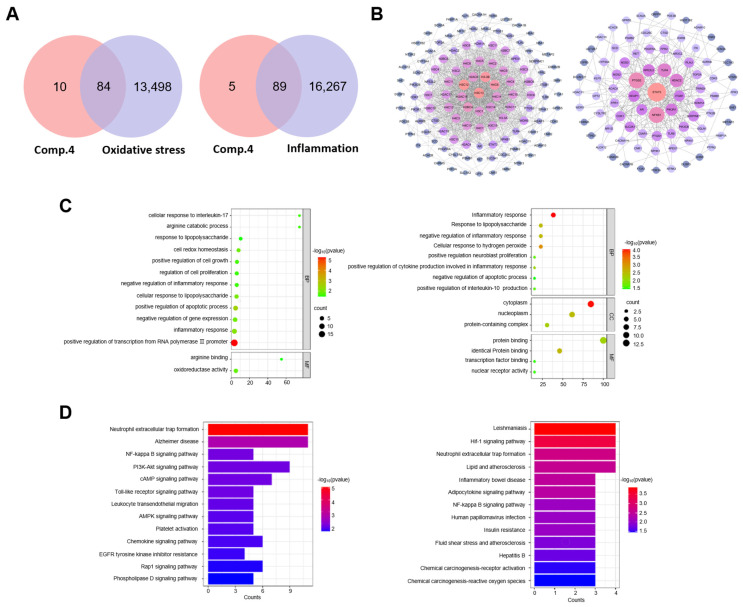
(**A**) Venn diagram illustrating the potential anti-oxidative stress and anti-inflammatory target genes of compound **4**. (**B**) Protein–protein interaction (PPI) network of target genes analyzed using STRING. (**C**) Bubble plot showing GO functional analysis of potential targets. (**D**) Bar plot of Kyoto Encyclopedia of Genes and Genomes (KEGG) pathway analysis.

The intersection of the 94 compound **4**-related target genes with oxidative stress- and inflammation-related disease target genes resulted 84 and 89 potential target genes, respectively (Figure 5A). The potential target genes were imported into the STRING database to construct a protein–protein interaction (PPI) network. The STRING results were then visualized and analyzed using Cytoscape software (version 3.10.2) to generate a network of potential target genes (Figure 5B).

GO enrichment analysis indicated 269 oxidative stress-related and 90 inflammation-related terms. Figure 5C displays the 14 significantly enriched terms associated with oxidative stress and 15 significantly enriched terms associated with inflammation. The enriched biological process terms primarily included negative regulation of inflammatory response, positive regulation of IL-10 production, and cellular response to H_2_O_2_. Molecular function terms were primarily involved in arginine binding, oxidoreductase activity, and transcription factor binding. The cellular component terms were predominantly associated with protein-containing complexes and the cytoplasm.

KEGG pathway enrichment analysis (Figure 5D) identified 26 inflammation-related signaling pathways, with significant enrichment observed for pathways related to chemical carcinogenesis-receptor activation, TLR signaling pathway, NF-κB signaling pathway, and AD were significantly enriched. The NF-κB signaling pathway, along with its upstream and downstream pathways associated with inflammation and oxidative stress, was significantly enriched in both network pharmacology and transcriptomics KEGG analyses. This suggests that the potential mechanism through which compound **4** alleviates H_2_O_2_-induced cellular damage may involve the NF-κB signaling pathway.

### 2.6. Compound ***4*** Attenuates H_2_O_2_-Induced Oxidative Stress Damage in PC12 Cells

**Figure 6:**
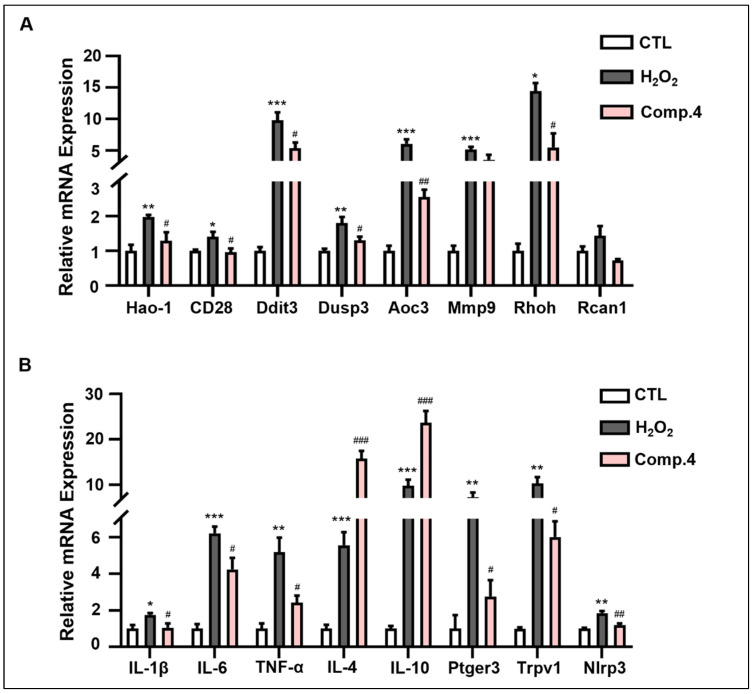
(**A**) Validation of oxidative stress-related mRNA screened using transcriptomics real-time quantitative reverse transcription polymerase chain reaction (qRT-PCR). (**B**) Validation of inflammation-related mRNA screened by transcriptomics using qRT-PCR. * Indicates significant difference (* *p* < 0.05, ** *p* < 0.01, *** *p* < 0.001) between control and H_2_O_2_ or compound–H_2_O_2_ groups. # represents a significant difference (# *p* < 0.05, ## *p* < 0.01, ### *p* < 0.001) between H_2_O_2_ and compound–H_2_O_2_ groups.

To validate the transcriptomics results, the mRNA levels of oxidative stress-related genes were measured. As shown in Figure 6A, relative to the control group, the mRNA levels of pro-oxidant genes, namely *HAO-1*, *CD28*, *DDIT3*, *DUSP3*, *AOC3*, *MMP9*, and *RHOH*, were upregulated in the model group (*p* < 0.01 or *p* < 0.001). However, following treatment with compound **4**, the expression levels of *HAO*-*1*, *CD28*, *DDIT3*, *DUSP3*, and *AOC3* were downregulated (*p* < 0.05 or *p* < 0.01), while *MMP9* and *RHOH* expression showed a decreasing trend, albeit without statistical significance. These findings indicate that compound **4** mitigates H_2_O_2_-induced oxidative stress damage in PC12 cells by reversing the upregulation of pro-oxidant gene expression induced by H_2_O_2_.

To further validate the transcriptomics results, the expression levels of inflammation-related genes were measured (Figure 6B). Compared to the control group, the H_2_O_2_-induced model group exhibited significant upregulation in the expression of both pro-inflammatory factors (including IL-1β, IL-6, TNF-α, PTGER3, TRPV1, and NLRP3) and anti-inflammatory factors (IL-4 and IL-10) (*p* < 0.05, *p* < 0.01, or *p* < 0.001). However, treatment with compound **4** downregulated the expression of IL-1β, IL-6, TNF-α, PTGER3, TRPV1, and NLRP3, and further upregulated the expression of anti-inflammatory factors, IL-4 and IL-10, compared to that in the model group (*p* < 0.05 or *p* < 0.01). These results indicate that compound **4** mitigated H_2_O_2_-induced inflammatory damage in PC12 cells by modulating the expression of inflammatory factors.

### 2.7. Mechanism of Compound ***4*** in Ameliorating H_2_O_2_-Induced Neuronal Damage in PC12 Cells

**Figure 7:**
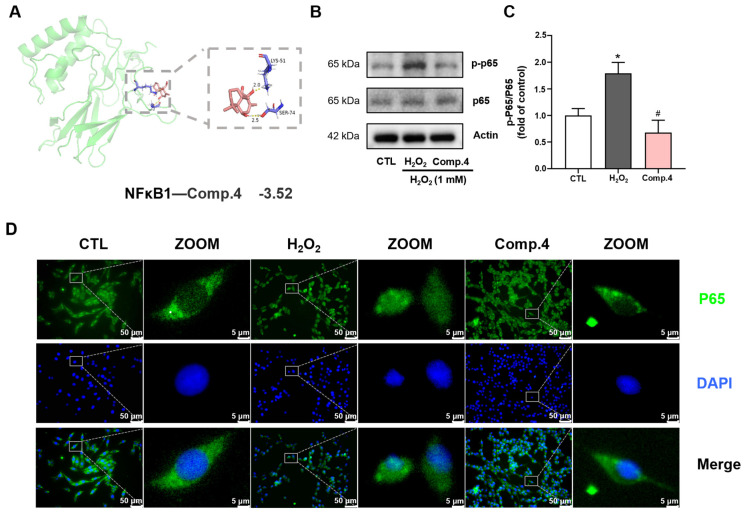
(**A**) Molecular docking of the three-dimensional (3D) model of the NF–κB1 protein with the crystal structure of compound **4**. (**B**) The protein expression of p-P65/P65 (*n* = 3). (**C**) The quantitative analysis of the signal densities of p65/p-p65. (**D**) Immunofluorescence staining of NF-κB P65. Data are expressed as mean ± SEM. Significance was calculated using a one-way ANOVA followed by Dunnett’s post hoc test. * Indicated significant difference (* *p* < 0.05) between control and H_2_O_2_ groups. # represented a significant difference (# *p* < 0.05) between H_2_O_2_ and compound **4**–H_2_O_2_ groups. Color code: Blue: DAPI (nuclear stain), green: P65 (target protein), black: merged channels.

Molecular docking of compound **4** with the NF-κB1 protein was performed (Figure 7A). Compound 4 formed two hydrogen bond interactions with the NF–κB1 receptor target via amino acid residues LYS-51 and SER-74, with a binding energy of −3.52 kcal/mol. These results suggest a strong binding affinity between compound **4** and the NF–κB1 protein, indicating that NF–κB1 may be a potential target of compound **4**.

Network pharmacology and transcriptomic analyses indicated marked alterations in the NF-κB signaling pathway. H_2_O_2_ stimulation substantially increased the expression of phosphorylated p65 (p-p65), whereas treatment with compound **4** markedly suppressed p-p65 levels (Figure 7B,C). To further validate the impact of compound **4** on the NF-κB signaling pathway, the role of NF-κB p65 was investigated using immunofluorescence staining analysis of p65 expression in PC12 cells to further validate the impact of compound **4** on the NF-κB signaling pathway (Figure 7D). H_2_O_2_ stimulation triggered the nuclear translocation of p65, whereas treatment with compound **4** inhibited this translocation. In summary, our findings demonstrate that compound **4** inhibits the activation of the NF-κB pathway in H_2_O_2_-induced PC12 cells, indicating that compound **4** may exert neuroprotective effects by alleviating H_2_O_2_-induced cellular damage through the NF-κB pathway.

## 3. Discussion

This study is the first to report a novel terpenoid compound, compound **4**, isolated from the fruiting bodies of *H. erinaceus*. Its neuroprotective activity was validated through various experiments, including network pharmacology prediction, transcriptomic analysis, immunofluorescence staining, and qRT-PCR. In an H_2_O_2_-induced neuronal injury model, compound **4** substantially attenuated oxidative stress and inflammatory responses in cells. Although previous studies have demonstrated that other components of *H. erinaceus*, such as polysaccharides, primarily exert neuroprotective effects by modulating immune cell function [25], the terpenoid compound identified in this study likely influenced redox balance and inflammatory responses in neuronal cells through specific signaling pathways.

Terpenoids, structurally diverse secondary metabolites composed of isoprene units and classified (e.g., monoterpenes, sesquiterpenes, diterpenes) based on unit count, play a key role in macrofungal bioactivity. Particularly, diterpenoids demonstrate potent neurotrophic factor synthesis-inducing activity [26,27], while the broader class demonstrates potent antioxidant activity through effective reactive oxygen species (ROS) scavenging and free radical quenching in essential botanical extracts [28]. Notably, diterpenoids represent the most intensively studied subclass, with most displaying marked neurotrophic activity. Zhao et al. [29] isolated 20 novel diterpenoids from *Pimpinella candolleana* 12 conferring substantial neuroprotection. Similarly, Li et al. [30] isolated five novel triterpenoids from *Ganoderma cochlear*, two suppressing inflammation in LPS-induced RAW264.7 cells. Mirroring these observations, the terpenoid compound **4** identified in the present study also exhibited anti-inflammatory and antioxidant activities, holding significant therapeutic potential for the development of neuroprotective agents targeting the mitigation of neuronal damage mediated by oxidative stress and neuroinflammation.

This study indicated substantial enrichment of the IL-17, TNF, and TLR signaling pathways. These pathways convergently activate the IKK complex, including IκBα degradation and nuclear translocation of NF-κB, thereby activating the NF-κB signaling pathway, which critically regulates inflammatory and immune responses [31,32,33]. Collectively, these results support the hypothesis that compound **4** exerts neuroprotective effects by modulating the NF–κB signaling pathway.

NF-κB is a critical transcription factor [34] that persists in an inactive cytoplasmic form under physiological conditions by binding to its inhibitory protein, IκB [35]. Upon stimulation with H_2_O_2_, IκB undergoes phosphorylation and degradation, releasing the p50 (NF-κB) and p65 (NF-κB1) heterodimer. The p65 subunit subsequently migrates to the nucleus and, in conjunction with p50, regulates the transcription of pro-inflammatory and antioxidant enzyme genes, thereby maintaining the intracellular redox balance [36,37].

In the current study, the results from the PC12 cell model suggest that compound **4** may mitigate neurodegenerative pathologies by suppressing NF-κB activation and nuclear translocation, thereby ameliorating oxidative stress and inflammation. Therefore, these findings highlight the potential of compound **4** as a therapeutic strategy to modulate the NF–κB pathway in oxidative stress- and inflammation-associated neurological disorders.

The PC12 cell line, a well-established neuroendocrine model, is extensively utilized to generate in vitro models of neurological disorders and physiological processes for probing the underlying mechanisms of natural small-molecule compounds. For instance, Li et al. [38] demonstrated that tetrahydroberberine, isolated from *Corydalis* (Papaveraceae), attenuated lipid peroxidation and inhibited ferroptosis in both a mouse model of spinal cord injury and an RSL3-induced PC12 ferroptosis model by activating the Nrf2 signaling pathway, thereby promoting neuronal survival. Similarly, Chen et al. [39] established that resveratrol suppressed ferroptosis via the AKT/NRF2 pathway in both a chronic unpredictable mild stress (CUMS) rat depression model and an erastin-induced PC12 ferroptosis model, alleviating depressive-like behaviors. Su et al. [40] identified a novel 2-arylbenzo[b]furan-4-vinylcarbonyl derivative of salvianolic acid C (6p) that exhibited neuroprotective effects in tert-Butyl hydroperoxide (t-BHP)-injured PC12 cells and middle cerebral artery occlusion (MCAO) rats via NRF2-mediated upregulation of the antioxidant enzyme HO-1. Additionally, Wang et al. [41] found that Eupalinolide B (EB), a bioactive compound isolated from *Eupatorium lindleyanum* DC, mitigated corticosterone (CORT)-induced PC12 cell damage and alleviated depressive-like behaviors in CUMS rats by modulating the GSK-3β/β-catenin pathway. Collectively, these studies highlight that natural small-molecule compounds exert neuroprotective effects through diverse signaling pathways, offering therapeutic potential in neurological diseases.

To date, the etiology of neurological disorders remains unclear, and their complex pathogenesis has resulted in the lack of targeted and effective targeted therapies [42]. Although natural small-molecule compounds derived from *H. erinaceus* have exhibited neuroprotective properties and may aid in the prevention or treatment of neurodegenerative diseases, research on their mechanisms of action and pharmacodynamics remains limited [17]. The novelty of this study resides in its complementary integration of network pharmacology, transcriptomics, and experimental validation to explore the neuroprotective activity and mechanisms of action of a novel monoterpenoid isolated from *H. erinaceus*. This approach enhances our understanding of intricate biological processes and offers new avenues for medicinal and functional food applications of *H. erinaceus*.

Certain limitations warrant acknowledgment. First, this work exclusively employed cell models to validate the neuroprotective effects and mechanisms of compound **4**, and there may be discrepancies between cellular and in vivo results [43]. Second, the research only focused on the NF–κB signaling pathway, without fully exploring the specific interactions between compound **4** and molecular components within this pathway. Other signaling pathways may also contribute to the neuroprotective effects of compound **4**, warranting further investigation.

## 4. Materials and Methods

### 4.1. General Information

HRESIMS spectra were recorded on an Agilent 6210 TOF LC/MS. IR spectra were obtained on a Nexus 870 FT-IR spectrometer. NMR spectra were recorded on a Bruker AV-500 NMR spectrometer. Melting points were determined on a Boetius micro-melting-point apparatus and were uncorrected. Single-crystal X-ray diffraction data were collected on an Agilent Technologies SuperNova Dual diffractometer with an Atlas detector (Cu Kα radiation, λ = 1.54184 Å). Optical rotations were recorded on a Rudolph Autopol III automatic polarimeter. UV spectra were recorded on a Hitachi U-3000 spectrophotometer. Silica gel (200−300 mesh; Qingdao Marine Chemical Factory, Qingdao, China) and Sephadex LH-20 gel (Pharmacia Biotech, Uppsala, Sweden) were used for column chromatography (CC). HPLC was performed with a Hitachi L-7110 pump and L-7400 UV detector equipped with an Apollo C18 column (5 μm, 250 mm × 4.6 mm; Alltech Associates, Inc. Chicago, IL, USA).

### 4.2. Fungal Material

Mature *H. erinaceus* fruiting bodies, procured from Nanjing Qingliang Edible Fungus Co., Ltd. (Nanjing, China), were mechanically pulverized, sieved (100-mesh), and then lyophilized to obtain the final dried powder.

### 4.3. Extraction and Isolation

*H. erinaceus* fruiting body powder (5 kg) underwent maceration with 95% ethanol (m/v 1:1). After filtration, the ethanol phase was concentrated in vacuo (55 °C, ≤100 mbar) to yield a syrupy extract. The ethanol extract was mixed with water and subjected to extraction with an equal volume of ethyl acetate. The combined organic layers were dehydrated and concentrated under vacuum at 55 °C until complete evaporation, yielding a crude extract (17 g).

The ethyl acetate fraction was separated by silica gel column chromatography. Initial elution was performed with 8 L of pure petroleum ether, followed by gradient elution with 9 L of petroleum ether-ethyl acetate mixtures at ratios of 95:5, 90:10, 80:20, and 50:50. Subsequently, 9 L of pure ethyl acetate was used for elution, followed by gradient elution with 9 L of ethyl acetate-methanol mixtures at ratios of 19:1 and 9:1. Finally, pure methanol was used for complete elution. The eluates were collected in 500 mL fractions using conical flasks, concentrated by rotary evaporation, and combined into six fractions (Fr.1: 1.2 g; Fr.2: 4 g; Fr.3: 4 g; Fr.4: 3 g; Fr.5: 2 g; Fr.6: 3 g). Silica gel (350 g, 200–300 mesh) was used for column chromatography with a gradient elution of CH_2_Cl_2_/MeOH (*v*/*v* 100:0, 100:1, 100:2, 100:4, 100:8, 100:20, 0:100, 3.5 L each), monitored by thin-layer chromatography (TLC). Fraction 3 was further purified on a Sephadex LH-20 column using methanol as the eluent and subsequently separated by preparative HPLC (Apollo C18 column (5 μm, 250 × 4.6 mm); isocratic methanol-water (65:35, *v*/*v*) at 2.0 mL/min) to yield colorless crystals of compounds **1** (21 mg), **3** (17 mg), **4** (20 mg), and **5** (25 mg). Fraction 4 was further separated by ODS column chromatography using methanol as the eluent, yielding colorless crystals of compound **2** (13 mg).

### 4.4. Cell Culture

The PC12 cell was provided by Professor Jianmei Li’s laboratory at the School of Food Science and Pharmaceutical Engineering, Nanjing Normal University. PC12 cells were maintained at 37 °C under 5% CO_2_ humidified conditions, using high-glucose DMEM culture medium enriched with 10% FBS and 1% penicillin-streptomycin antibiotic solution.

### 4.5. Cell Viability

PC12 cells were seeded in 96-well plates (3.0 × 10^5^ cells/well) and acclimatized in DMEM for 24 h. Test groups received fresh medium containing compound **4** (0.01 to 0.5 mg/mL) for 24 h, 100 μL cytotoxicity was assessed by adding Cell Counting Kit-8 (CCK-8; C0040, Beyotime Biotechnology, Shanghai, China) dissolved in PBS followed by 20 min at 37 °C. Absorbance at 450 nm (reference 650 nm) was measured using a microplate reader.

### 4.6. Transcriptome Experiment and Analysis

Transcriptomic profiling of the control and model group PC12 cells was conducted via RNA sequencing (RNA-seq). RNA-seq libraries were prepared and sequenced on the OE Biotechnology Co., Ltd. (Shanghai, China) platform, with four samples collected per group. Total RNA isolated with Trizol reagent underwent poly(A) selection using oligo(dT) magnetic beads. Complementary DNA (cDNA) synthesis was performed after the total RNA fragment was fragmented into short fragments and the messenger RNA (mRNA) was enriched using oligonucleotide (dT) magnetic beads.

We implemented differential expression analysis via DESeq2, identifying differentially expressed genes (DEGs) under threshold criteria of |log_2_FC| ≥ 0.58 (fold-change ≥ 1.5) and adjusted *p*-value ≤ 0.05. Visualization of DEGs utilized the OECloud platform (https://cloud.oebiotech.com/, (accessed on 1 November 2024)) to generate volcano plots and hierarchical clustering heatmaps. Functional annotations of DEGs included Gene Ontology (GO) enrichment and Kyoto Encyclopedia of Genes and Genomes (KEGG) pathway analysis while gene set enrichment was assessed using Gene Set Enrichment Analysis (GSEA v4.1.0) with hallmark gene sets (FDR < 0.25). The GSEA software was obtained from the Broad Institute (Cambridge, MA, USA).

### 4.7. Network Pharmacology Analysis

#### 4.7.1. Clustering of Compound **4** and Oxidative Stress-Related Target Genes

A network pharmacology approach elucidated compound **4**’s neuroprotective mechanisms. Target genes associated with compound **4** were retrieved from the SuperPred database (https://prediction.charite.de/ (accessed on 29 October 2024)) based on its chemical structure, yielding 94 potential target genes. Disease-related target genes for oxidative stress and inflammation were mined from the GeneCards and OMIM databases (https://www.omim.org/ (accessed on 29 October 2024)), resulting in 13,582 and 16,356 target genes, respectively. UniProt standardized the gene symbols (https://www.uniprot.org (accessed on 29 October 2024)). The intersection of disease-related target genes and compound **4** target genes was identified using Venny 2.1 software (https://bioinfogp.cnb.csic.es/tools/venny/ (accessed on 29 October 2024)), revealing potential therapeutic targets. The potential target genes were subsequently imported into the STRING database to construct a protein–protein interaction (PPI) network. Cytoscape software (version 3.10.2) performed topological analysis to generate a network of the highest-confidence subnetwork.

#### 4.7.2. Protein–Protein Interaction (PPI) Network Map of Potential Target Genes

Compound 4’s oxidative stress-related targets were analyzed using the STRING database (https://string-db.org (accessed on 29 October 2024)), with *Homo sapiens* species restriction and default parameters. The resulting PPI network, encompassing co-expression, gene fusion, neighborhood, and co-localization evidence types, was constructed and imported into Cytoscape software (version 3.10.2) for topological analysis. Nodes represent proteins while edges indicate functional associations with evidence codes.

### 4.8. GO and KEGG Pathway Enrichment Analysis

Gene Ontology (GO) functional enrichment and Kyoto Encyclopedia of Genes and Genomes (KEGG) signaling pathway enrichment analysis was performed using the DAVID database (https://davidbioinformatics.nih.gov/ (accessed on 29 October 2024)). The top 13 pathways with *p* value ≤ 0.05 and Benjamin value ≤ 0.05 were selected in KEGG biological pathway enrichment analysis. GO enrichment analysis included biological process (BP), cellular component (CC), and molecular function (MF). The bioinformatics platform (https://www.bioinformatics.com.cn/ (accessed on 29 October 2024)), a learning platform for biological information analysis, was used to visualize the results and clarify the main pharmacological mechanism of compound **4** affecting OS.

### 4.9. ROS Staining

Intracellular ROS levels were quantified using DCFH-DA (Beyotime Biotechnology, Shanghai, China). After exposure to the indicated dose of compound **4**, cells were PBS-washed and loaded with DCFH-DA working solution for 20 min (37 °C, dark). Cells were observed under an inverted fluorescence microscope and the positive stained area was measured using ImageJ win-64 software.

### 4.10. SOD Assay

SOD activity was quantified using SOD Assay Kit (Beyotime Biotechnology, Shanghai, China). PC12 cells were seeded in a six-well plate. After treatment, cells were pretreated according to the experimental methodology provided by the kits. Then, the protein concentration was determined by a BCA assay kit. Next, according to manufacturer’s protocol, we set the blank hole, standard aperture, measure hole, and control hole, and added the SOD working solution in the cell detection plate. After that, we incubated cells for 30 min at 37 °C. Finally, absorbance was measured at 450 nm.

### 4.11. Quantitative Real-Time PCR

PC12 cells were seeded in 12-well plates at 2 × 10^5^ cells/well. Total RNA extraction employed Trizol reagent (Himedia Laboratories, LLC, Kelton, PA, USA) under ambient conditions (25 °C), with purity and quantity verified by Nanodrop (Thermo Scientific 2000c, Waltham, MA, USA). cDNA synthesis utilized 1 μg total RNA via reverse transcription. The list of primers used are described in Table 2. Quantitative PCR amplification implemented Cham Q SYBR Master Mix (Vazyme Biotechnology, Nanjing, China) with gene-specific primers (Table 2) under standardized cycling conditions as follows: 95 °C/30 s, 40 cycles of 95 °C/5 s and 60 °C/30 s.

### 4.12. Molecular Docking

Target protein crystal structures retrieved from the RCSB Protein Data Bank (https://www.rcsb.org (accessed on 30 October 2024)) underwent PyMOL 2.2.0 (Schrodinger LLC, New York, NY, USA) preprocessing to remove water and ligands), add polar hydrogens, and assign Gastiger charges. Compound 4’s 3D molecular structure was obtained by the PubChem database (https://pubchem.ncbi.nlm.nih.gov/ (accessed on 30 October 2024)) and saved in SDF format. The SDF file was converted to MOL2 format using OpenBabel 3.1.1 software (http://openbabel.org/wiki/Main_Page (accessed on 30 October 2024)). Molecular docking was performed using AutoDock 4.2.6 software (The Scripps Research Institute, La Jolla, CA, USA), with the protein-ligand complex exhibiting the lowest binding energy selected as the optimized target. The docking results were visualized and analyzed using PyMOL 2.2.0.

### 4.13. Western Blotting

Cell lysates were prepared in ice-cold RIPA buffer containing protease and phosphatase inhibitors and centrifuged at 12,000 rpm at 4 °C for 10 min using a centrifuge (Sorvall Legend Micro 21R, Thermo Fisher Scientific, Waltham, MA, USA). Protein quantification employed a bicinchoninic acid protein assay kit (E112-02, Vazyme, Nanjing, China) against bovine serum albumin standards. Equal protein amounts of each sample were separated by sodium dodecyl sulfate-polyacrylamide gel electrophoresis and then transferred onto polyvinylidene fluoride membranes (ISEQ00010, Millipore, Bedford, MA 01730, USA). After 2 h blocking with 5 % skimmed milk, membranes were incubated overnight at 4 °C with primary antibodies and then incubated with HRP-conjugated secondary antibodies (1 h RT). Primary antibodies included rabbit anti-P-NFκB p65 (WL01273b, Wanlei Life Sciences, Shenyang, China), rabbit anti-NFκB p65 (WL02169, Wanlei Life Sciences), and mouse anti-β-actin (T0022, Affinity Biosciences, Sydney, Australia). Protein bands were visualized using a Tanon-5200 Chemiluminescence Imager (180-501, Tanon Science & Technology Co., Ltd., Shanghai, China). The density of bands was quantified using ImageJ (Version win64, National Institutes of Health, Bethesda, MD, USA), normalized to internal reference protein and expressed as fold change relative to the control value.

### 4.14. Immunofluorescence (IF) Staining

Cell monolayers underwent fixation with 4% paraformaldehyde (PFA) (30 min, RT), permeabilization in 0.1% Triton X-100/PBS, and blocking with QuickBlock^TM^ (Dundee, Scotland) Buffer (10 min). Primary anti-NF-κB/p65 antibody incubation proceeded overnight at 4 °C. After PBS washes, samples received Nestin488-conjugated secondary fluorescent antibodies (ThermoFisher, Waltham, MA, USA) and DAPI (4′,6-diamidino-2-phenylindole) nuclear counterstain. All images within experimental sets were acquired under identical exposure parameters using an inverted fluorescence microscope (Olympus, Tokyo, Japan), with intensity histograms monitored to prevent saturation.

### 4.15. Statistics Analysis

All experiments included 2–3 independent biological replicates. Statistical analyses utilized Graphpad Prism 8.0.1, employing two-tailed unpaired t-tests for between-group comparisons. The results are detailed in the figures and the figure legends. Sample sizes were determined based on experience from previous work and based on other similar published studies. Quantitative results are presented as mean ± SEM in figures, with full statistical details annotated in figure legends.

## 5. Conclusions

**Figure 8:**
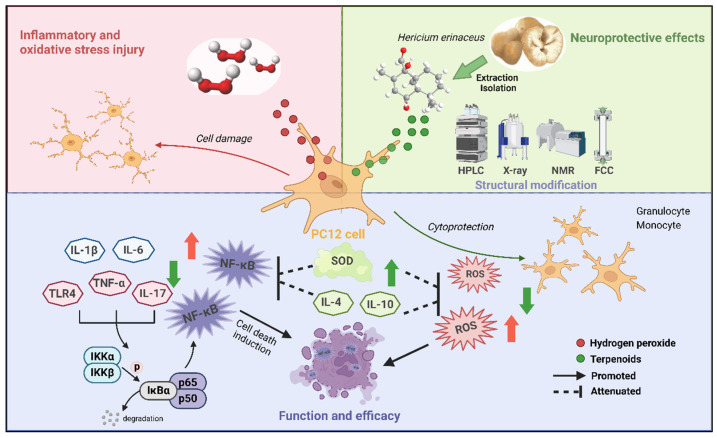
Schematic illustration of the proposed neuroprotective mechanism of terpenoid compound 4 from *H. erinaceus* fruiting bodies via the NF-κB signaling pathway (this figure was created using BioRender (https://biorender.com/). Color code: Red arrows: expression changes induced by H_2_O_2_ treatment; Green arrows: expression modulation after compound **4** administration. Copyright permissions for the use of the platform’s visualization tools have been obtained).

This study identifies a novel neuroprotective terpenoid (4) from *H. erinaceus* fruiting bodies. Through a multi-omics-guided strategy (transcriptomics, network pharmacology, molecular docking) and experimental verification, compound **4** attenuated H_2_O_2_-induced nerve injury by suppressing oxidative stress and inflammation through the NF-κB signaling pathway (Figure 8). These findings position *H. erinaceus* as a promising therapeutic source for neurodegenerative disorders. Current limitations, specifically the exclusive use of in vitro models and underexplored pathway crosstalk such as Nrf2/ARE, direct future research toward in vivo validation in Aβ-mouse models and multi-target mechanism elucidation for enhanced clinical translation.

## Figures and Tables

**Table 1:** ^1^H (500 M Hz, CDCl_3_) and ^13^C NMR (150 M Hz, CDCl_3_) data for Compound **4**.

Pos.	δH	δC	Pos.	δH	δC
1	1.46 (1H, m), 1.50 (1H, m)	43	9		82.6
2	1.54 (1H, m), 1.72 (1H, m)	33.9	10		43.5
3	1.28 (1H, m), 1.37 (1H, m)	18.1	-CHO	9.79 (H, s)	205.9
4		33.3	12	1.60 (3H, d, *J* = 1.7 Hz)	36.3
5	1.64 (1H, d, *J* = 2.4 Hz)	49.8	13	1.12 (3H, s)	23
6	4.28 (1H, dt, *J* = 10.2, 2.4 Hz)	70	14	1.29 (3H, s)	18.1
7	5.83 (1H, dq, *J* = 10.2, 1.7 Hz)	133.8	15	1.18 (3H, s)	18.5
8		130.9			

**Table 2:** Sequences and of the primers used for RT-qPCR.

Gene ID	Forward Primer (5′–3′)	Reverse Primer (5′–3′)
Aoc3	CCAGCAGTTCTAGCATCTACAACC	CCAAGTCCTCGCCAGCAATG
CD28	TTGGCTCTCAGCTTCTTCTCAG	GTAAAGGGATGCCCGGAACT
Ddit3	ATCTTCATACACCACCACACCTG	TAGGGATGCAGGGTCAAGAGT
Dusp3	AGGCAGAATCGTGAGATCGG	TTCCTAAGTAGGGCAGCCAG
Hao-1	ACCTCACTGCCCATTGTTGTAAAG	TAAGATCCCATCCACACCATGTTTAAC
IL-1β	TCAGACAGCACGAGGCATTT	AGCTTCAGGAAGGCAGTGTC
IL-4	GTACCAGACGTCCTTACGGC	TCAGACCGCTGACACCTCTA
IL-6	CACTTCACAAGTCGGAGGCT	TCTGACAGTGCATCATCGCT
IL-10	TTGAACCACCCGGCATCTAC	CCAAGGAGTTGCTCCCGTTA
Mmp9	CAAACCCTGCGTATTTCCATTCATC	GATAACCATCCGAGCGACCTTTAG
Nlrp3	TGGACCTCAACAGACGCTACAC	GTCCTGCCAATGGTCAAGAGTTC
Ptger3	GCAATTCCTTCCTAATCGCCG	AGGTTGTTCATCATCTGGCA
Rcan1	GCCCTTCGCACCCTTCTCC	CACCTCCTCCATCTCGCAGTC
Rhoh	AGGCAGATGTGGTACTAATGTGTTAC	TCCTGACCTCACTAATCCATTTGTTC
TNF-α	GGAGGGAGAACAGCAACTCC	GCCAGTGTATGAGAGGGACG
Trpv1	TTATGTTCGTCTACCTCGTGTTCTTG	CATAGGCAGAGAGTTATTCTTCCCATC

## Data Availability

Data are contained within the article and Supplementary Materials.

## Supplementary Materials

The following supporting information can be downloaded at: https://www.mdpi.com/article/10.3390/ijms26146606/s1.

## Abbreviations

The following abbreviations are used in this manuscript:

AD	Alzheimer’s disease
COSY	^1^H–^1^H correlation spectroscopy
CUMS	Chronic unpredictable mild stress
DEGs	Differentially expressed genes
GO	Gene Ontology
GSEA	Gene Set Enrichment Analysis
HRMS	High-resolution mass spectrometry
KEGG	Kyoto Encyclopedia of Genes and Genomes
MCAO	Middle cerebral artery occlusion
NGF	Nerve growth factor
NMR	Nuclear magnetic resonance
NO	Nitric oxide
PCA	Principal component analysis
PPI	Protein–protein interaction
ROS	Reactive oxygen species
SOD	Superoxide dismutase
TLR	Toll-like receptor
TNF-α	Tumor necrosis factor
XRD	X-ray diffraction
