# Supplementary material for: Neuroprotective Terpenoids Derived from Hericium erinaceus Fruiting Bodies: Isolation, Structural Elucidation, and Mechanistic Insights
Source: Int J Mol Sci. 2025 Jul 10;26(14):6606. doi: 10.3390/ijms26146606 (PMC12294627; doi:10.3390/ijms26146606)
Supplement: Supplementary file 1 [file ijms-26-06606-s001.zip › ijms-3732880-supplementary.pdf]

SUPPLEMENTARY MATERIALS include Supplementary Figures S1-S6

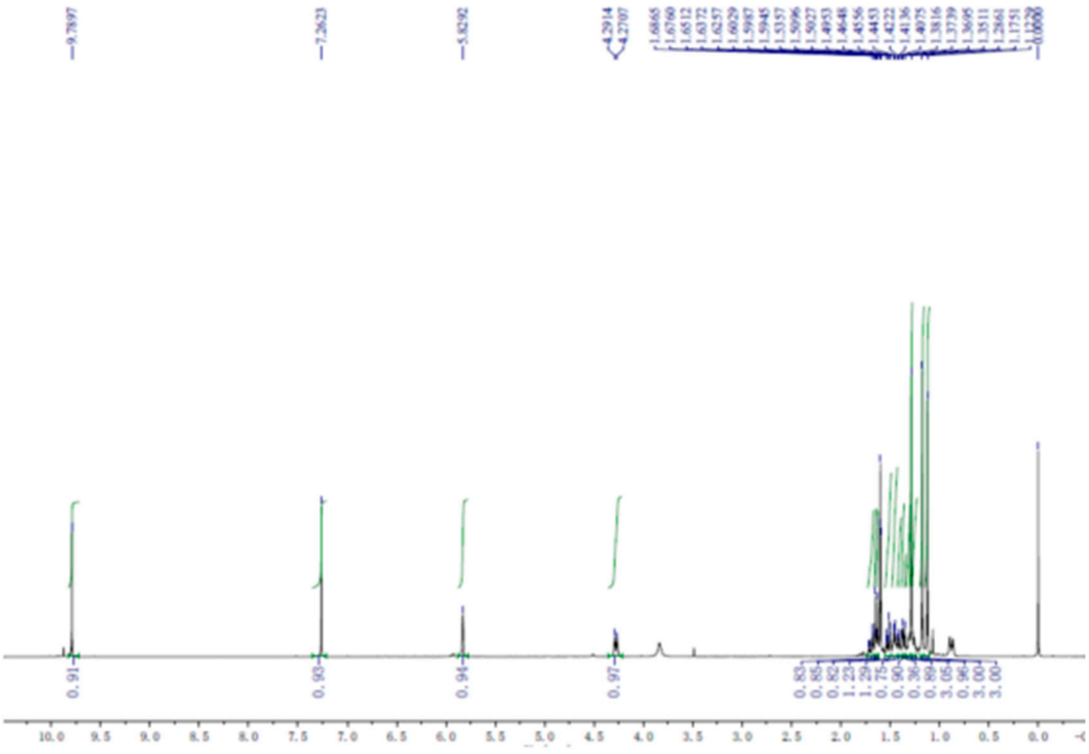

Supplementary Figure S1. ^{1}H spectra of compound 4 in CDCl3. (500 MHz).

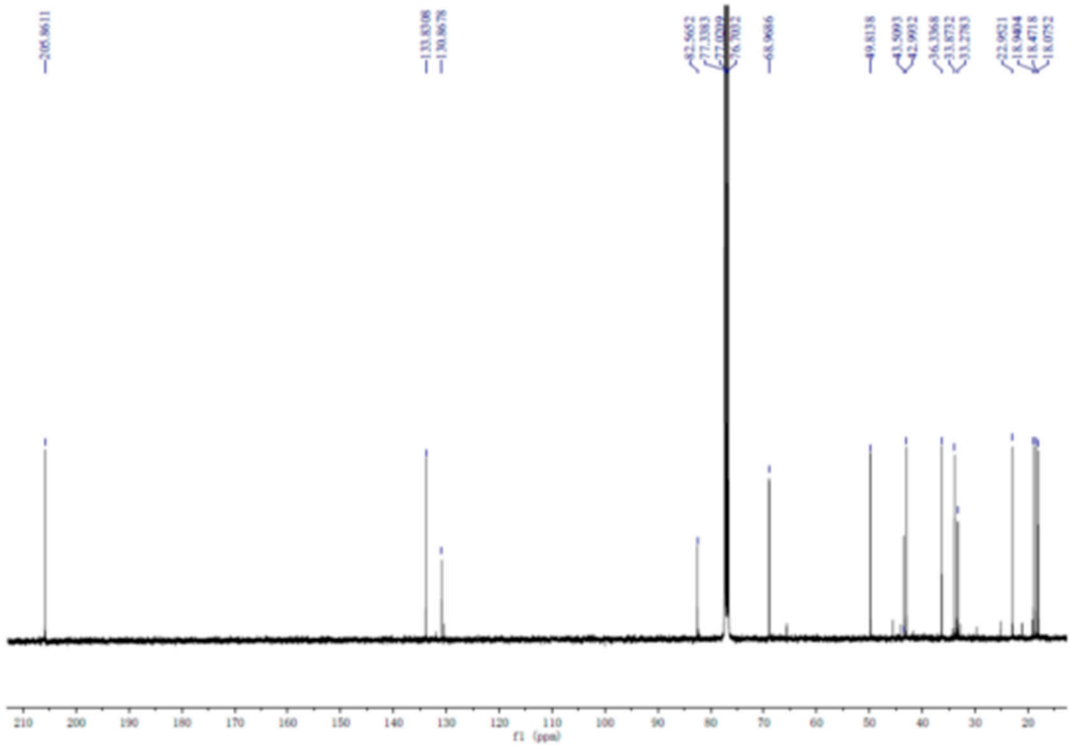

Supplementary Figure S2.^{13}C spectra of compound 4 in CDCl3. (150 MHz).

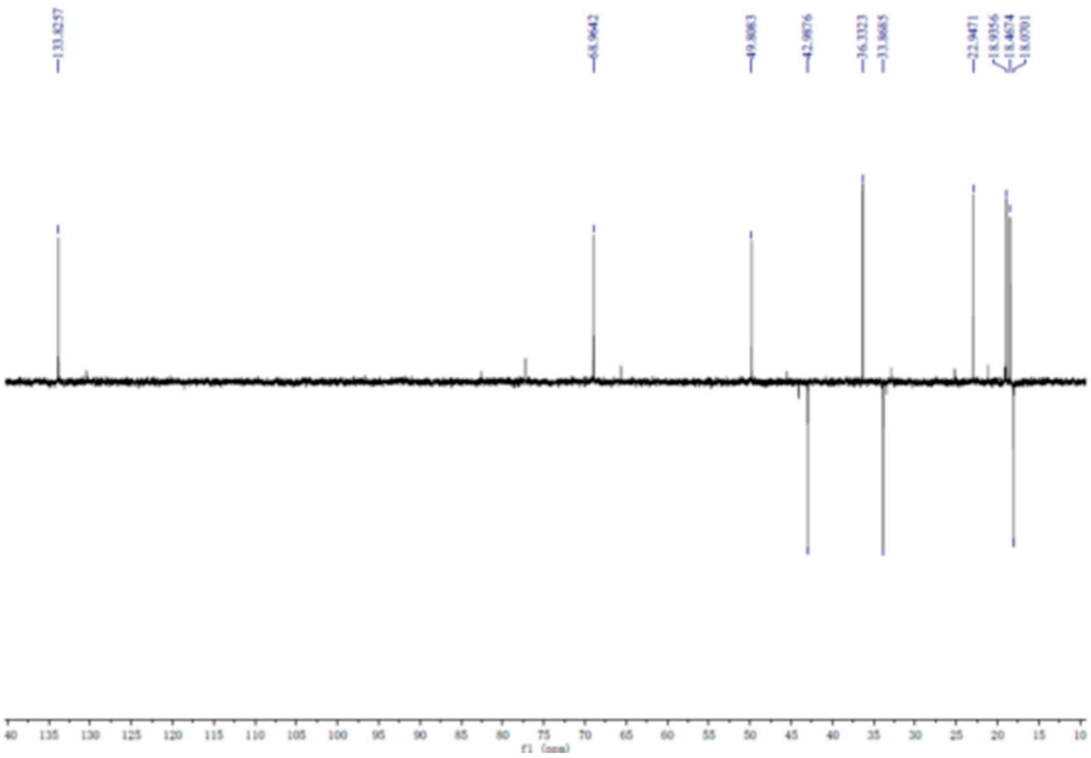

Supplementary Figure S3. DEPT-135 spectrum of compound 4 in CDCl3 (125 MHz).

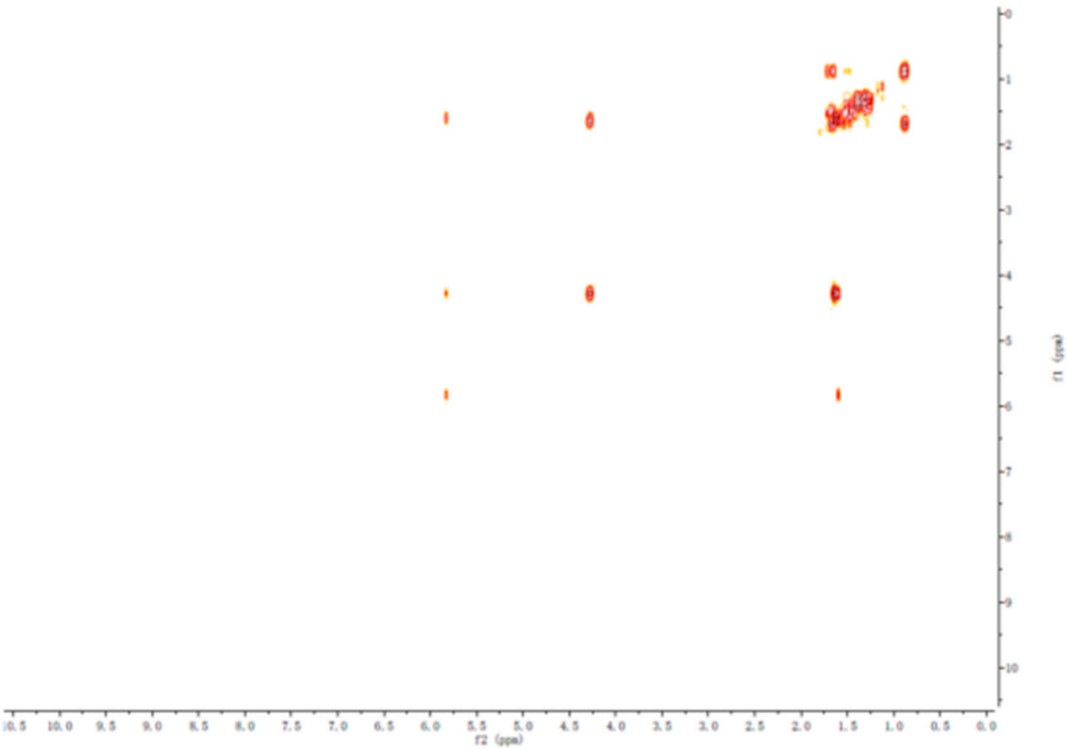

Supplementary Figure S4. ^1H-^1H COSY spectrum of compound 1 in CDCl3 (500 MHz).

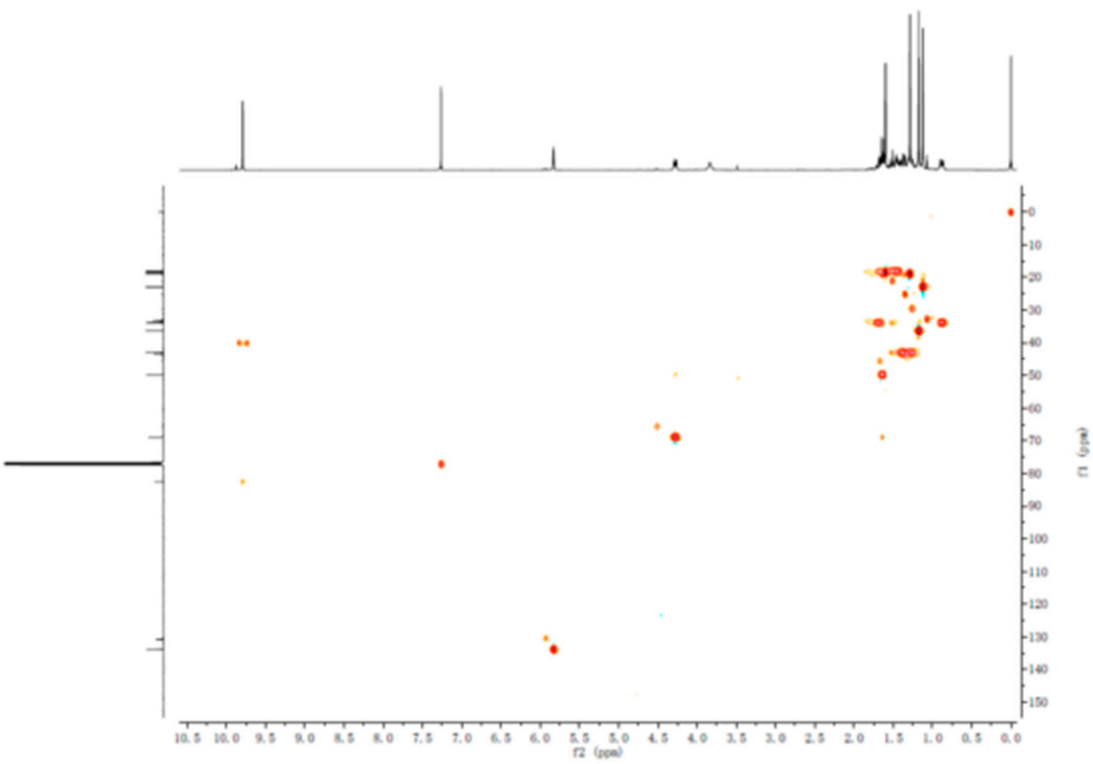

Supplementary Figure S5. HSQC spectrum of compound 1 in CDCl3 (500 MHz).

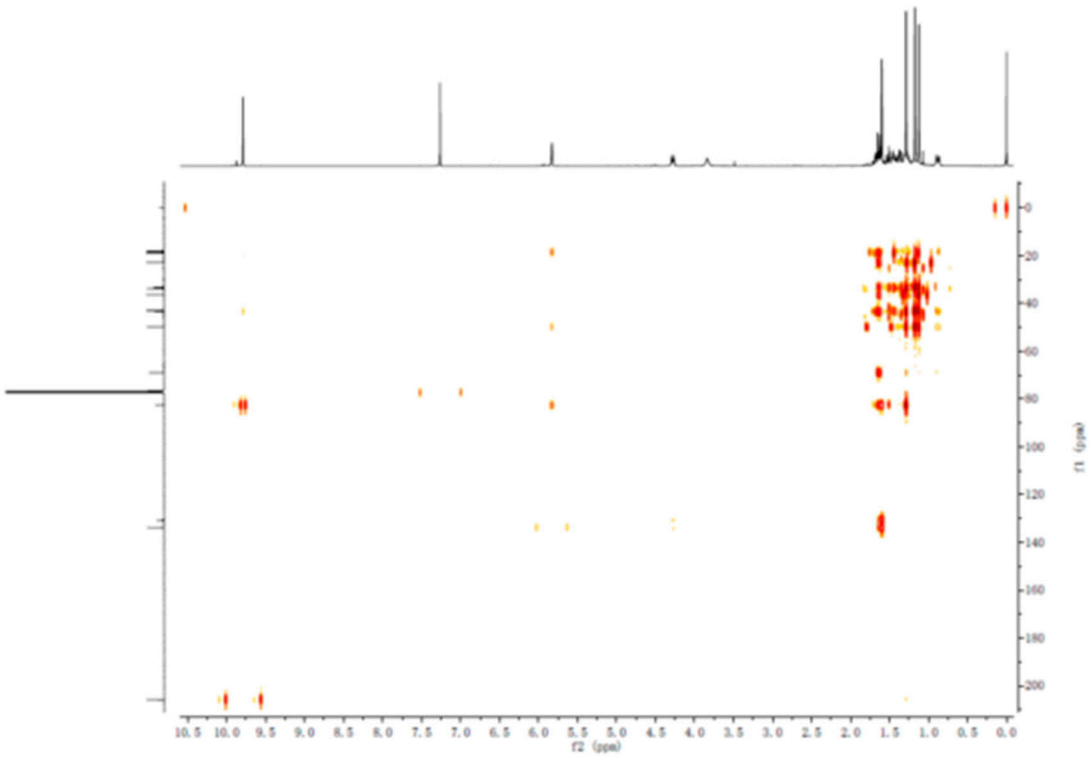

Supplementary Figure S6**.** HMBC spectrum of compound 1 in CDCl3 (500 MHz).
